# Nachwuchsmangel in der Thoraxchirurgie: Müssen wir uns anpassen oder aussterben?

**DOI:** 10.1007/s00104-024-02106-w

**Published:** 2024-05-31

**Authors:** Romina Maria Rösch, Raffaella Griffo, Josephine Berger-Groch, Lena Brendel, Maria Ada Presotto, Isabella Metelmann, Hauke Winter, Laura Valentina Klotz

**Affiliations:** 1 Abteilung für Thoraxchirurgie, Thoraxklinik Heidelberg, Röntgenstr. 1, 69126 Heidelberg, Deutschland; 2 Deutsches Zentrum für Lungenforschung – Zentrum für Translationale Lungenforschung (TLRC), Universitätsklinikum Heidelberg, Neuneheimer Feld 130.3, 69120 Heidelberg, Deutschland; 3 Abteilung für Orthopädie, Rehaklinik Sonnhalde, Am Schellenberg 1, 78166 Donaueschingen, Deutschland; 4 Abteilung für Pneumologie, Thoraxklinik Heidelberg, Röntgenstr. 1, 69126 Heidelberg, Deutschland; 5 Klinik und Poliklinik für Viszeral-, Transplantations-, Thorax- und Gefäßchirurgie, Universitätsklinikum Leipzig, Liebigstraße 20, 04103 Leipzig, Deutschland

**Keywords:** Thoraxchirurgie, Medizinstudium, Chirurgische Weiterbildung, Ausbildung, Umfrage, Thoracic surgery, Medical studies, Surgical training, Education, Survey

## Abstract

Hintergrund: Obwohl die Thoraxchirurgie ein anspruchsvolles und vielseitiges chirurgisches Fachgebiet ist, wird in den kommenden Jahren ein Mangel an qualifizierten und motivierten Assistenzärzten für die Thoraxchirurgie erwartet. Es wird mit einem Mangel von ca. 7300 Chirurgen in der stationären Versorgung gerechnet. Daher ist es dringend erforderlich, mehr interessierte junge Medizinstudierende zu gewinnen und die medizinische Ausbildung unserer nächsten Generation von Chirurgen zu verbessern.

Methoden: Eine Onlineumfrage mit 39 Fragen zur Demografie der Teilnehmer, medizinischen Ausbildung, zum Interesse an der chirurgischen Ausbildung und der thoraxchirurgischen Weiterbildung sowie zur Attraktivität der Facharztausbildung wurde erstellt, um den aktuellen bundesweiten Status quo unter Medizinstudierenden zu evaluieren.

Ergebnisse: Insgesamt konnten 224 Fragebögen zur Auswertung herangezogen werden. Grundsätzlich zeigte sich zu Beginn des Studiums ein hohes Interesse an der (Thorax-)Chirurgie. Hervorzuheben ist, dass ein Drittel nicht wusste, dass der „Thoraxchirurgische-Facharzt“ ein eigenständiger Facharzt ist. Diese Aussage wirft weitere Fragen bzgl. der Präsenz der Thoraxchirurgie im Medizinstudium auf. Gefragt nach typischen Eigenschaften, die die Studierenden mit der Thoraxchirurgie verbinden, wurde zum Großteil mit „einer hohen praktischen Tätigkeit“ geantwortet. Was sie von einer chirurgischen Weiterbildung abhält, wurde vorrangig mit der schlechten Vereinbarkeit von Familie und Beruf begründet.

Schlussfolgerung: Die Studierenden wissen genau, was sie sich für ihre Zukunft wünschen und wo die Chirurgie ihre Schwachpunkte hat. Sie wünschen sich eine transparente und praxisorientierte Weitbildung, die Vereinbarkeit von Familie und Beruf sowie die Anerkennung ihrer Arbeit und Person.

## Abstract (en)

Background: Although thoracic surgery is a challenging and versatile surgical specialty, a shortage of qualified and motivated thoracic surgery residents is expected in the coming years. In the inpatient setting, a shortage of approximately 7300 surgeons is expected. Therefore, there is an urgent need to attract more interested young medical students and improve the medical training of our next generation of surgeons.

Methods: To assess the current nationwide status quo among medical students, an online survey with 39 questions on participant demographics, medical education, interest in surgical and thoracic surgery training, and attractiveness of residency was designed.

Results: In all, 224 questionnaires were analyzed. Overall, there was a high level of interest in (thoracic-) surgery at the start of training. It should be noted that one third of the respondents did not know that the ’thoracic surgeon’ is an independent specialist. This statement raises further questions about the presence of thoracic surgery in medical studies. When asked about typical characteristics that students associate with thoracic surgery, the majority answered ’a high level of practical activity’. The main reason they gave for not pursuing further surgical training was the unfavorable work-life balance.

Conclusion: Students know exactly what they want for their future and where surgery has its weaknesses. They want transparent and practical training, a work-life balance, and recognition of their work and themselves.

## Hintergrund

Innovative und qualitativ hochwertige Medizin basiert auf einer strukturierten und fundierten Ausbildung der jungen Generation. Die Chirurgie ist wie die meisten medizinischen Berufe jedoch von einem relevanten Nachwuchsproblem betroffen. Aufgrund des demografischen Wandels erwarten Studien im Jahr 2035 knapp 100.000 unbesetzte ärztliche Stellen in Deutschland [1, 2]. Die Gründe hierfür sind vielfältig. Einer wird in der Feminisierung der Medizin gesehen. Im Wintersemester 2021/2022 waren zwei Drittel der Studierenden in der Humanmedizin Frauen [1]. Aufgrund von Kindererziehungszeiten und Care-Tätigkeiten arbeiten Frauen oft in Teilzeit und bevorzugt in nichtchirurgischen Disziplinen. Des Weiteren führt die oft hohe Arbeitsbelastung zu einer Abwanderung der Kolleginnen und Kollegen in patientenferne Bereiche wie Verwaltung, Management oder Forschung. Wie in allen anderen chirurgischen Fächern zeichnet sich daher auch in der Thoraxchirurgie ein Mangel an qualifizierten und motivierten Ärztinnen und Ärzten in Weiterbildung ab.

Um einen objektiven Überblick über die aktuelle Situation der Studierenden in Deutschland zu erhalten, wurde eine Umfrage unter den Medizinstudierenden an deutschen Universitäten durch das „Junge Forum der Deutschen Gesellschaft für Thoraxchirurgie“ (Junges Forum der DGT) und der „Arbeitsgemeinschaft Frauen in der Thoraxchirurgie“ (AG FiT) durchgeführt.

Ziel dieser Umfrage war es, das Image und die Potenziale einer chirurgischen Weiterbildung mit Schwerpunkt auf die Thoraxchirurgie unter Medizinstudierenden in Deutschland zu eruieren. Im Speziellen wurde die Zufriedenheit mit der studentischen Lehre, die Kenntnis des Faches Thoraxchirurgie, das Vorhandensein allgemeiner Vorurteile sowie mögliche Verbesserungspotenziale evaluiert.

## Methodik

Die anonymisierte Umfrage wurde mit dem Onlineumfragesystem LimeSurvey (LimeSurvey GmbH Umfragedienste & Beratung, Hamburg, Deutschland) erstellt und im Zeitraum von Februar bis April 2022 online durchgeführt. Die Zugangsdaten für die Teilnahme an der Umfrage wurden per Mail an alle Fachschaften für Medizin der Universitäten in Deutschland, mit der Bitte um Weiterleitung an alle Studierenden der Medizin, versandt.

Der Fragebogen wurde in Zusammenarbeit von Junges Forum der DGT und der AG FiT erstellt. Insgesamt wurden 39 Fragen zu den demografischen Daten der Studierenden, zum generellen chirurgischen Interesse, zur Lehre in der Thoraxchirurgie und zur Attraktivität des Fachgebiets in der Umfrage entworfen. Im Rahmen der Umfrage wurden zudem konkrete Möglichkeiten zur Attraktivitätssteigerung evaluiert. Die Antworten konnten je nach Fragentyp anhand einer fünfteiligen Skala oder als nominale Antwort gegeben werden. Ferner bestand die Möglichkeit, Kommentare in Freitextform anzugeben.

Die Auswertung erfolgte mit den Statistikfunktionen von Microsoft Excel für Mac (Microsoft Redmond, WA, USA) und Numbers (Apple Inc. Cupertino, CA, USA). Die demografischen Daten wurden deskriptiv zusammengestellt. Kontinuierliche Variablen wurden mittels Mittelwert und Standardabweichung aufgeführt. Kategoriale Variablen wurden mit Zahl und Prozentangabe dargestellt.

## Ergebnisse

Insgesamt nahmen 240 Studierende an der Umfrage teil. Insgesamt wurden 224 Fragebögen vollständig ausgefüllt und konnten für die weitere Auswertung herangezogen werden.

### Demografische Daten

Insgesamt 55 (25 %) der Befragten waren Medizinstudierende der Vorklinik, 141 (63 %) der Klinik und 28 (12 %) bereits im Praktischen Jahr (PJ). Im Geschlechterverhältnis zeigt sich, dass mehr als zwei Drittel (73 %) weiblich waren, 27 % männlich und eine Person gab an, keinem der zur Verfügung stehenden Geschlechter anzugehören. Das Alter betrug im Mittel 23,9 ± 3,6 Jahre.

Drei Prozent der Befragten waren zum Zeitpunkt der Umfrage bereits Eltern von durchschnittlich zwei Kindern (2 ± 0,8). 214 der Befragten (96 %) waren ledig, 9 (4 %) verheiratet und ein Studierender war alleinerziehend.

Insgesamt hatten 58 Befragte (26 %) bereits eine Ausbildung vor dem Medizinstudium abgeschlossen, 88 % davon in einem medizinischen Bereich und der Rest in einem nichtmedizinischen Feld. Weitere demografische Daten sind in Tab. 1 aufgeführt.

**Tab. 1:** Demografische Daten der Medizinstudierenden

Demografische Daten	*n* (%); M ± SD
*Weiblich*	162 (72 %)
*Männlich*	61 (27 %)
*Divers*	1 (1 %)
*Alter*	23,9 ± 3,6
*Vorklinik*	55 (25 %)
*Klinik*	141 (62 %)
*PJ*	28 (13 %)
*Ledig*	214 (95 %)
*Verheiratet*	9 (4 %)
*Alleinerziehend*	1 (1 %)
*Eltern*	6 (3 %)
Männlich	1 (0,5 %)
Weiblich	5 (2 %)
*Anzahl der Kinder*	2 (2 ± 0,8)
*Haben Sie vor dem Studium bereits eine Ausbildung abgeschlossen?*	
Ja (gesamt)	58 (26 %)
Ja, medizinischer Bereich	51 (23 %)
Ja, nichtmedizinischer Bereich	7 (3 %)
Nein	166 (74 %)

### Chirurgische Weiterbildung

Die Frage nach dem Interesse an einer chirurgischen Weiterbildung wurde von 78 Befragten (35 %) mit „Ja“ beantwortet, 73 Studierende (33 %) waren unschlüssig und 73 Studierende (33 %) hatten zum Zeitpunkt der Umfrage kein Interesse an einer chirurgischen Weiterbildung im Anschluss an das Medizinstudium (Tab. 2).

**Tab. 2:** Interesse an einer (thorax-)chirurgischen Weiterbildung und Erfahrungen mit der Thoraxchirurgie

Frage	*n* (%)
*Haben Sie Interesse an einer chirurgischen Weiterbildung?*	
Ja	78 (35 %)
Eventuell	73 (33 %)
Nein	73 (33 %)
*Haben Sie Interesse an einer thoraxchirurgischen Weiterbildung?*	
Ja	17 (8 %)
Eventuell	76 (34 %)
Nein	131 (58 %)
*Wissen Sie, dass der „Thoraxchirurgische Facharzt“ ein eigenständiger Facharzt ist?*	
Ja	153 (68 %)
Nein	71 (32 %)
*Hatten Sie bereits die Möglichkeit, bei einem operativen Eingriff in der Thoraxchirurgie teilzunehmen?*	
Nein	117 (52 %)
Ja, aber ich hatte kein Interesse	5 (2 %)
Ja, als Zuschauer	72 (32 %)
Ja, als Assistenz	30 (13 %)
*Wünschen Sie sich mehr Lehrveranstaltungen zur Thoraxchirurgie?*	
Nein	81 (36 %)
Ja	143 (64 %)
– Ja, in Form von Seminaren	69 (31 %)
– Ja, in Form von Vorlesungen	15 (7 %)
– Ja, in Form von Wahlfächern	59 (26 %)

Gefragt nach dem expliziten Interesse an einer thoraxchirurgischen Weiterbildung, antworteten 17 Studierende (8 %) mit einem sehr hohen Interesse, 76 (34 %) waren unentschlossen und 131 (58 %) hatten kein Interesse an einer thoraxchirurgischen Weiterbildung. Hervorzuheben ist, dass etwa ein Drittel der 224 Befragten nicht wusste, dass der Facharzt für Thoraxchirurgie ein eigenständiger Facharzt ist (Tab. 2).

Die Lehrveranstaltungen der Thoraxchirurgie finden nach Angabe von 197 Studierenden (88 %) an den Universitätskliniken statt. Bei einem thoraxchirurgischen Eingriff haben 107 Studierende (58 %) zum Zeitpunkt der Umfrage bereits im Operationsaal zugeschaut oder assistiert. Insgesamt wünschen sich 143 Studierende (64 %) mehr Lehrveranstaltungen in der Thoraxchirurgie (Tab. 2).

Basierend auf den Angaben der Studierenden zeigte sich im Laufe des Studiums ein deutlicher Abfall des Interesses an der Chirurgie und Thoraxchirurgie. So hatten 42 % der Studierenden in der Vorklinik Interesse an der Chirurgie, im PJ waren es nur noch 36 %. Analog hierzu zeigten 13 % der Studierenden in der Vorklinik Interesse an der Thoraxchirurgie, während im PJ kein Interesse mehr bestand (Tab. 3).

**Tab. 3:** Interesse an der Chirurgie/Thoraxchirurgie nach Ausbildungsstand (Vorklinik, Klinik und PJ)

	Vorklinik (%)	Klinik (%)	PJ (%)
Interesse an der Chirurgie	42	33	36
Interesse an der Thoraxchirurgie	13	8	0

### Was hält Sie von einer chirurgischen Weiterbildung ab?

**Abb. 1:**
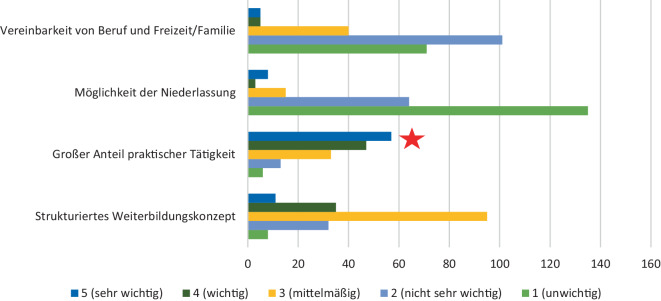
Was verbinden Sie mit der Thoraxchirurgie (auf einer Skala von 1 [unwichtig] bis 5 [sehr wichtig])? Der *rote Stern* markiert den, laut Umfrageteilnehmer und -teilnehmerinnen, relevantesten Punkt

**Abb. 2:**
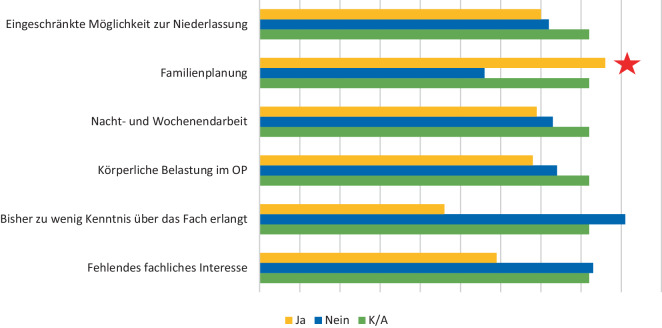
Was hält Sie von einer chirurgischen Weiterbildung ab? Der *rote Stern* markiert den, laut Umfrageteilnehmer und -teilnehmerinnen, relevantesten Punkt

Der am häufigsten genannte Faktor, weshalb sich die Studierenden gegen eine chirurgische Weiterbildung entscheiden, stellt die Familienplanung bzw. die Work-Life-Balance dar. Weitere genannte Faktoren waren die eingeschränkte Möglichkeit der Niederlassung, die Nacht- und Wochenendarbeit sowie die körperliche Belastung im Operationssaal. Tatsächlich ist der hohe praktische Anteil von den Studierenden am häufigsten genannte Faktor, der mit der Thoraxchirurgie in Verbindung gebracht wird (Abb. 1). Ein mangelndes Wissen über die Thoraxchirurgie oder über die entsprechende Weiterbildung sowie ein fehlendes fachliches Interesse stellten keine Gründe dar, weshalb sich die Umfrageteilnehmer(innen) gegen eine chirurgische oder thoraxchirurgische Weiterbildung entscheiden würden (Abb. 2).

**Abb. 3:**
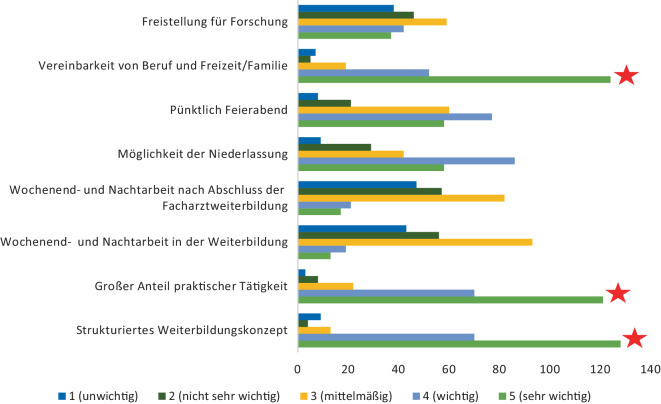
Welche Aspekte sind Ihnen für eine Facharztweiterbildung wichtig? Die *roten Sterne* markieren die, laut Umfrageteilnehmer und -teilnehmerinnen, relevantesten Punkte

Für die Studierenden steht im Rahmen der Facharztweiterbildung daher die Vereinbarkeit von Familie und Beruf sowie Freizeitaktivitäten an erster Stelle, gefolgt von einer strukturierten Weiterbildung mit einem hohen praktischen Anteil (Abb. 3). Die Einhaltung der Arbeitszeiten, Nachtdienste und Wochenendarbeit wurden nur nachrangig genannt. Keine wesentliche Relevanz wurde dem Aspekt der Freistellung für Forschung gewidmet.

### Verbesserung der Weiterbildung

**Abb. 4:**
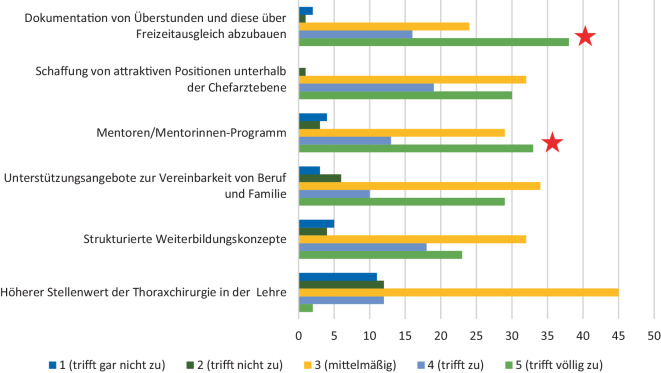
Was könnten Verbesserungsmöglichkeiten sein? Die *roten Sterne* markieren die, laut Umfrageteilnehmer und -teilnehmerinnen, relevantesten Punkte

Als spezifische Verbesserungsideen zeigten zwei Aspekte einen besonders hohen Stellenwert für die Studierenden. Die transparente Zeiterfassung mit Dokumentation der Überstunden und Kompensation durch Freizeitausgleich sowie die Implementierung eines Mentoringprogramms sind für die Studierenden die relevantesten Optimierungsstrategien für eine attraktive chirurgische Weiterbildung (Abb. 4).

Des Weiteren sind attraktive Positionen unterhalb der Chefarztebene sowie Unterstützungsangebote zur Vereinbarkeit von Familie und Beruf für die Studierenden von großer Relevanz.

Zuletzt konnten die Studierenden ihre Vorschläge zur Steigerung der Attraktivität einer Facharztweiterbildung in der Chirurgie mit Bezug auf die studentische Lehre, Weiterbildung und Arbeitsbedingungen frei formulieren. Zusammenfassend wünschen sich die Studierenden eine transparente, faire und vor allem praxisorientierte Weiterbildung, die eine Vereinbarkeit von Familie und Beruf erlaubt.

### Freitextantworten

Bei der letzten Frage wurde den Umfrageteilnehmerinnen die Möglichkeit gegeben, frei zu formulieren, was ihrer Meinung nach zum Attraktivitätsverlust der Chirurgie/Thoraxchirurgie führt und welche möglichen Verbesserungen in der Lehre/Weiterbildung und den Arbeitsbedingungen dazu beitragen können, die Attraktivität wieder zu steigern.

#### Zitate

„Regelmäßige ‚Auffrischungs-Seminare‘ für Ärzte! *Transparenz* in der Ausbildung zum Facharzt (wie viel kann ich wirklich mitmachen, werde ich angeleitet, darf ich bei OPs wirklich assistieren etc.).“

„Viele praktischen Tätigkeiten. Bessere *Work Life Balance*.“

„Mich würde überzeugen, wenn ich wüsste, dass ich als Assistenzärztin nicht *unterschätzt* werde, nur weil ich eine *Frau* bin. Es würde mich überzeugen, wenn ich wüsste, dass ich als *Thoraxchirurgin meine Arbeitszeiten einigermaßen mit der Familie kombinieren könnte, ohne ein Burnout zu entwickeln.“*

„Bessere Vereinbarkeit von *Freizeit und Beruf*!“

„*Ausgewogener Arbeitsalltag und Wertschätzung *der Person.“

„Es wäre gut, wenn eine Art ‚Einstellungstest‘ durchführt würde, um zu prüfen, ob ein gewisses chirurgisches Talent vorhanden ist.“

„Ich wurde bereits während einer Famulatur von FA-Weiterbildung für TCH überzeugt. Wichtig war dabei die Selbstwirksamkeitserfahrung (ich durfte im OP viel assistieren, auf Station eigene Patienten betreuen und war insg. wie ein Assistenzarzt ins Team eingebunden). Danach setzte dann die Überlegung ein zu Punkten wie* ‚Möglichkeit der Niederlassung‘, ‚Belastung in Diensten‘, ‚Vereinbarkeit von Familie und Beruf‘.“*

„Vereinbarkeit von *Familie und Beruf* z. B. halbtags arbeiten können, *Frauen-freundliches Umfeld*, flache Hierarchien, junges, dynamisches Team, keine Überstunden.“

## Diskussion

Die vorliegende Umfrage unter Medizinstudierenden zur Attraktivität einer Facharztausbildung in der Chirurgie und der Thoraxchirurgie im Speziellen zeigt einen Verlust der Attraktivität im Laufe des Studiums der Humanmedizin durch die Verschiebung verschiedener Motivationsfaktoren während des Studiums und die Eindrücke der Studierenden während ihrer Praktika im Rahmen der studentischen Lehre. Für eine effiziente Rekrutierung von Nachwuchs in der Chirurgie müssen die thoraxchirurgischen Abteilungen und die Fachgesellschaft ihre Startvorteile gegenüber anderen Fächern dringend und effizient optimieren. Als Gründe für den Attraktivitätsverlust gelten die auch in dieser Umfrage genannten Kriterien der schlechten Work-Life-Balance und hohen Arbeitsbelastung mit fehlender Flexibilität zur Vereinbarkeit von Freizeitaktivitäten bzw. von Familie und Beruf. Die genannten Kriterien werden durch die Studierenden bereits in ihren Famulaturen, Praktika und später im PJ wahrgenommen. In der studentischen Lehre ist die Thoraxchirurgie an den meisten Universitätskliniken unterrepräsentiert. Des Weiteren wird die theoretische und praktische Lehre in der Chirurgie aufgrund der knappen Zeitressourcen der Weiterbildenden häufig an junge Kollegen und Kolleginnen weitergereicht, die didaktisch und fachlich nicht ausreichend qualifiziert sind. Hierdurch kann unter den Studierenden der Eindruck entstehen, dass auf ihre Aus- und Weiterbildung nicht genügend Wert gelegt wird. Immerhin wünschen sich ca. 64 % der Befragten mehr Lehrveranstaltungen im Bereich der Thoraxchirurgie. Dieses Interesse an chirurgischen Fächern sollte früh gefördert werden. Daher haben einige Krankenhäuser, Fachgesellschaften und Junge Foren verschiedene Maßnahmen ergriffen, um dem Interessenverlust im Laufe des Studiums entgegenzuwirken. Hierbei werden mittlerweile unterschiedliche Konzepte wie beispielsweise Summer Schools und die Einführung von Simulationstraining basierend auf Virtual Reality und Robotik angeboten und stetig weiterentwickelt [3, 4].

Die Bundesvertretung der Medizinstudierenden in Deutschland (bvmd) stellte fest, dass bislang nur wenige Imagekampagnen für die Chirurgie durchgeführt wurden. Der ärztliche Beruf und die Chirurgie genießen in der Allensbacher Berufsprestige-Skala ein hohes Ansehen [5]. Die oben aufgeführten Zahlen und die beschriebenen Entwicklungen unterstreichen die Wichtigkeit einer strukturierten Weiterbildung und der Lehre [3–5]. Auch Schneider et al. beschrieben in ihrer Umfrage aus dem Jahr 2020 das hohe Grundinteresse an einer chirurgischen Facharztweiterbildung zu Studienbeginn [6]. Auch sie betonten diesen Vorteil zu anderen Disziplinen, jedoch setzen die vielfältigen Anstrengungen zur Rekrutierung von Nachwuchs in der Chirurgie meist erst gegen Ende des Studiums ein. Dabei haben sich zur langfristigen Nachwuchsakquise insbesondere frühzeitige Programme mit „Hands-on“-Training im chirurgischen Kernarbeitsbereich – dem Operationssaal – als erfolgreich erwiesen [6].

Seit Jahren belegen statistische Daten, dass die Humanmedizin weiblich wird [7]. Jedoch zeigen die aktuellen Daten der Bundesärztekammer von 2021, dass unter den 40.194 Fachärzten und -ärztinnen der Chirurgie nur 9162 (22,8 %) Chirurginnen sind.

Die Geschlechtsverteilung in der vorliegenden Umfrage mit einem Frauenanteil von 64,4 % spiegelt den bundesweiten Durchschnitt der Medizinstudierenden wider [8]. Daher muss das Berufsbild des Chirurgen und der Chirurgin dringend modifiziert werden. Männliche Rollenbilder sind immer noch vorherrschend, jedoch kann sich die heranwachsende Generation meist nicht mehr mit den oft veralteten Vorstellungen und Führungsstilen identifizieren. Um auch in Zukunft motivierte und engagierte Nachwuchschirurgen/-chirurginnen gewinnen zu können, müssen die Arbeitsbedingungen dringend dem Bedarf der jungen Mediziner und Medizinerinnen angepasst werden und gemeinsam Konzepte für eine mögliche Umsetzung in dem attraktiven Fachgebiet der Chirurgie erarbeitet werden.

Die Freitextantworten haben gezeigt, dass sich die Studierenden unabhängig des Geschlechts durchweg eine bessere Vereinbarkeit von Familie, Freizeit und Beruf wünschen. Durch die absolvierten Praktika im Rahmen der studentischen Lehre sollte die Thematik der Work-Life-Balance mit den Studierenden thematisiert werden, da in den letzten Jahren verschiedene Verbesserungen wie beispielsweise die Arbeitszeitregelung im Bereitschaftsdienst und die Vergütung von Arbeit an Wochenenden und Feiertagen gesetzlich geregelt wurden. Eine transparente und strukturierte Erfassung der Arbeitszeit ist auf Basis der gesetzlichen Arbeitszeitregelung im Rahmen der Tarifeinigung im Jahr 2019 flächendeckend verpflichtend. Nichtsdestotrotz müssen vor allem in den chirurgischen Fächern dringend Möglichkeiten der Weiterbildung in Teilzeit erarbeitet werden. Zudem hat bereits die europäische Umfrage von Pompili et al. gezeigt, dass Einrichtungen zur Kinderbetreuung im Krankenhaus den Arbeitsplatz der Chirurgie verbessern [9].

Weiterhin besteht in der chirurgischen Versorgung ein hoher Personalbedarf. Dieses Gap setzt sich einerseits aus der Überalterung der derzeit tätigen Chirurgen und Chirurginnen (sog. Ersatzbedarf) und andererseits aus der demografisch bedingten Zunahme der Patientenzahlen (sog. Mehrbedarf) zusammen [6]. Derzeit sind 30 % der ambulant und 11 % der stationär tätigen Chirurgen und Chirurginnen älter als 60 Jahre und nur ca. 2 % (ambulant) bzw. 21 % (stationär) der aktiv tätigen Kolleginnen und Kollegen 40 Jahre und jünger. Gleichzeitig sind die stationären chirurgischen Behandlungsfälle in den letzten Jahren um knapp 12 % gestiegen [6]. Wenn die sog. „Babyboomer“ in den nächsten Jahren in Rente gehen, wird sich dieser Personalmangel nochmals drastisch verschärfen. Dadurch wächst die neue Generation an Ärztinnen und Ärzten unter komplett konträren Bedingungen heran als die aktuelle Generation der Chefärzte und Chefärztinnen. Sie profitieren von dem vorherrschenden Fachkräftemangel, indem sie aus vielen Jobangeboten das zu ihren Lebensvorstellungen passende Angebot wählen können. Dadurch wachsen die Ansprüche an die Personalabteilung sowie an Chefärztinnen und Chefärzte [10–14].

Das Interesse an chirurgischen Fächern ist am Anfang des Medizinstudiums hoch. Neben den verbesserungswürdigen Arbeitsbedingungen sollte daher dringend an der Sichtbarkeit der Thoraxchirurgie bereits am Anfang des Studiums gearbeitet werden.

Das Alleinstellungsmerkmal des Operationssaals und die Faszination des Fachs mit seinen vielfältigen Möglichkeiten könnten zur Motivation hervorragend genutzt werden. Anhand der Umfragedaten zeigt sich, dass nur etwa die Hälfte der Teilnehmenden im Abschnitt der klinischen Ausbildung und zwei Drittel im Praktischen Jahr an einer thoraxchirurgischen Operation teilgenommen bzw. eine solche Operation gesehen haben. In einer Befragung von 482 Medizinstudierenden durch Sutton et al. konnte gezeigt werden, dass Praktika in chirurgischen Fächern besser bewertet werden als in anderen Fächern [15]. Daher sollten wir uns nicht, wie in anderen Studien gezeigt, auf die späteren Semester konzentrieren, sondern das hohe Interesse an chirurgischen Fächern nutzen und Studierende bereits früh durch Vorlesungen, Mentoringprogramme (wie bei dem Verein „Die Chirurginnen“) [16, 17] und Praktika für die Thoraxchirurgie motivieren. Um die Studierenden bereits frühzeitig in das chirurgische Netzwerk einzubinden, können diese beispielsweise eine kostenlose Mitgliedschaft im Verein Die Chirurginnen e. V. beantragen. Hierdurch kann die Begeisterung für die Chirurgie beispielsweise durch ein mittlerweile etabliertes Mentoringprogramm, Onlinevortragsreihen für junge Kolleginnen und Kollegen sowie niederschwellige Kommunikation in medizinischen Chats über das Studium hinaus gehalten werden.

Eine weitere Möglichkeit könnte die Übertragung von mehr Verantwortung unter Supervision im Rahmen der Facharztausbildung darstellen. Hierbei könnte ein bereits mehrfach geprüftes und für geeignet bewertetes „Train-the-Trainer“-Programm (TTT-Programm) eine gute Möglichkeit darstellen. Dieses Konzept zur Verbesserung der Lehrfähigkeiten von Ärztinnen und Ärzten ist weithin angenommen und evaluiert worden [18–22]. Der Vorteil für Assistenzärzte/-innen besteht nicht nur darin, dass sie ihr Wissen vertiefen und behalten, sondern gleichzeitig ihre Führungs- und Kommunikationsfähigkeiten weiterentwickeln, indem sie die erlernten Fähigkeiten weitergeben, entweder an jüngere Assistenten/-innen oder Studierende [23–25]. Das Unterrichten wurde als entscheidend für die Stärkung des Selbstvertrauens, der Motivation und des Enthusiasmus der Ärzte und Ärztinnen in Weiterbildung angesehen. In der Praxis führten diese Vorteile zu einer größeren Arbeitszufriedenheit, was wiederum die Produktivität der Ärzte und Ärztinnen in Weiterbildung und damit die Patientenversorgung und die klinischen Ergebnisse verbesserte [26, 27]. Dadurch kann den Studierenden ein gutes Konzept in der chirurgischen Lehre näher gebracht werden und gleichzeitig die Zufriedenheit der Ärztinnen und Ärzten in Weiterbildung gestärkt werden.

Zur besseren Vereinbarkeit von Familie, Freizeit und Beruf sollte eine Etablierung von Arbeitsmodellen in Teilzeit verpflichtend werden. Zudem sollte die Schaffung betrieblicher Krippen- und Kindergartenplätze, angepasst an die täglichen Arbeitszeiten, eine Selbstverständlichkeit sein.

Die beschriebenen Modelle mögen in Universitätskliniken oder in Kliniken mit Kontakt zu medizinischen Fakultäten leichter umzusetzen sein. Sutton et al. empfehlen, informelle Mentoringbeziehungen bereits zu Studierenden, Pflegepraktikanten und Hospitanten aufzubauen und über die gesamte Ausbildungszeit zu pflegen, da aus jedem Studierenden ein späterer Facharzt oder eine Fachärztin für Chirurgie werden kann [15]. Die Fachgesellschaften, Kliniken, Personalabteilungen und Chefärzte/-ärztinnen sollten das vorhandene Interesse der Studierenden an der Chirurgie und Thoraxchirurgie zu Beginn des Medizinstudiums zu ihrem Vorteil nutzen und durch die oben erwähnten Motivatoren gezielt halten, um den Nachwuchs in chirurgischen Fächern zu sichern.

## Fazit

- Es besteht die dringende Notwendigkeit, den chirurgischen Nachwuchs adäquat zu fordern und zu fördern, da ein Teil der Studierenden grundsätzlich bereit ist, eine chirurgische Laufbahn einzuschlagen, wenn die Voraussetzungen dafür gegeben sind.
- Die allgemeine Weiterbildungssituation und die Vereinbarkeit von Familie, Beruf und Freizeit sind entscheidende Stellschrauben, um eine erfolgreiche Nachwuchsakquise für die chirurgische und insbesondere die thoraxchirurgische Weiterbildung zu betreiben.

Einige der oben genannten Faktoren sind fremdbestimmt und können von Chirurginnen und Chirurgen nicht direkt beeinflusst werden. Allerdings sollten die Lehre und Präsenz unseres Fachgebietes vor allem an den Universitätskliniken gefördert werden. Unterstützungsangebote (z. B. Krippenplätze) zur Vereinbarkeit von Familie und Beruf stellen die Grundlage einer familienfreundlichen und zeitgemäßen Arbeitsatmosphäre dar und sind somit nicht nur dringend notwendig, sondern sollten auch niederschwellig angeboten werden.
